# Long-Term Radiographic Stability of Tibial Plateau Angle Following TPLO in Small-Breed Dogs: A Retrospective Study

**DOI:** 10.3390/ani16142112

**Published:** 2026-07-08

**Authors:** Dahyun Jeong, Joowon Ahn, Hwi-Yool Kim

**Affiliations:** 1 Department of Veterinary Surgery, Konkuk University, Seoul 05029, Republic of Korea; k9751009@gmail.com (D.J.); rffwon@naver.com (J.A.); 2 FM Animal Hospital, Gimpo-si 10080, Republic of Korea

**Keywords:** tibial plateau leveling osteotomy, small-breed dogs, tibial plateau angle, cranial cruciate ligament disease, long-term radiographic outcome

## Abstract

Cranial cruciate ligament disease (CCLD) is among the most common orthopedic conditions in dogs, and tibial plateau leveling osteotomy (TPLO) is the current standard of care; however, long-term radiographic data on tibial plateau angle (TPA) stability specific to small-breed dogs (<10 kg) remain limited. This retrospective study evaluated radiographic correction and long-term stability of TPA following TPLO in small-breed dogs and assessed whether observed TPA changes fell within the range of measurement error. Thirty stifles from 25 small-breed dogs (body weight 2.56–9.46 kg) that underwent TPLO between 2022 and 2025 were reviewed, with a mean follow-up of 12.6 ± 6.8 months. TPA was measured preoperatively, immediately postoperatively, at 1–2 months, and at ≥6 months using digital orthopedic planning software. Preoperative TPA averaged 31.23 ± 4.60° and decreased to 4.68 ± 1.84° immediately after surgery; at ≥6 months, mean TPA was 5.19 ± 1.83°. The mean change (ΔTPA) was 0.52 ± 0.64° (95% CI: 0.28–0.75°). Although statistically significant (*p* < 0.001), this fell below the 1.5° intraobserver measurement error previously reported. Body weight showed no correlation with ΔTPA (r = −0.059, *p* = 0.756). These findings were robust in a sensitivity analysis restricted to one stifle per dog (*n* = 25; mean ΔTPA 0.56 ± 0.67°) and in the subgroup without concurrent medial patellar luxation (*n* = 19; mean ΔTPA 0.40 ± 0.49°), both remaining within the reported measurement error. TPLO achieved effective and radiographically stable TPA correction in small-breed dogs.

## Simple Summary

Tearing of the cranial cruciate ligament in the knee is one of the most common causes of hindlimb lameness in dogs. A surgery called tibial plateau leveling osteotomy is widely used to treat it by changing the slope at the top of the shin bone. Most studies of how stable this slope remains after surgery have been performed in medium- and large-breed dogs, and little is known about small dogs weighing under 10 kg. In this study, we reviewed the radiographs of 30 knees from 25 small-breed dogs that underwent this surgery and followed them for an average of about one year. The corrected slope changed very little over time—on average less than half a degree—and this small change fell within the range of normal measurement error. A dog’s body weight was not related to the amount of change. These findings suggest that the surgery provides a reliable and durable correction in small-breed dogs and should reassure veterinarians and owners that the corrected angle remains stable on long-term radiographs.

## 1. Introduction

In dogs, cranial cruciate ligament disease (CCLD) ranks among the most frequent causes of hindlimb lameness, producing instability of the stifle, degenerative joint change, and reduced limb function. If treatment is not undertaken, the affected joint deteriorates in a progressive and irreversible manner [1,2].

Originally described by Slocum and Slocum [3], tibial plateau leveling osteotomy (TPLO) counteracts cranial tibial thrust by means of a proximal tibial osteotomy and rotation, with the goal of reducing the postoperative TPA to approximately 5–6° [4]. Clinical evidence has repeatedly demonstrated favorable results, establishing TPLO as the technique of choice for most canine patients [5,6,7].

Accurate measurement of the TPA underpins both surgical planning and the evaluation of postoperative results. Fettig et al. [8] reported radiographic measurement variability of roughly 0.8° between different observers and 1.5° for repeated measurements by the same observer. Inaccurate limb positioning during radiography may contribute a further 1–2° of variation [9]. These error margins must be kept in mind when postoperative TPA changes are interpreted, as a shift of under 1° is more plausibly explained by measurement variability than by a true biological change [8,10].

A progressive postoperative rise in TPA after TPLO has been documented in several reports [11,12]. The recent prospective study of Volz et al. [13], by contrast, found only a small mean TPA change of 0.22 ± 0.75° at 6 months—comfortably within measurement error—and detected no association between TPA change and functional outcome, although a lower TPA at 6 months corresponded to more symmetrical hindlimb gait.

The available literature on postoperative TPA change derives largely from medium- and large-breed dog populations. Small-breed dogs, however, are distinguished by lower body mass, smaller tibial dimensions, and lower absolute joint loading—features that could affect tibial remodeling after surgery [14,15]. Despite the growing use of TPLO in small-breed dogs, long-term radiographic data specific to this group remain scarce [14,16].

Against this background, the present study evaluated TPA correction and its postoperative stability in small-breed dogs (<10 kg), focusing on whether any observed TPA changes fell within previously published limits of measurement variability. We hypothesized that the TPA would remain stable, with any deviations too small to carry clinical significance.

## 2. Materials and Methods

### 2.1. Study Design and Patient Selection

For this retrospective review, the clinical records and radiographs of small-breed dogs (<10 kg) that had undergone TPLO at FM Animal Hospital (Gimpo-si and Goyang-si, Gyeonggi-do, Republic of Korea) between 2022 and 2025 were collected. Thirty stifles from 25 client-owned dogs satisfied the inclusion criteria. A portion of this dataset was previously presented in the first author’s Master’s thesis [17].

To be included, a case had to satisfy all of the following: a body weight below 10 kg, TPLO as the primary procedure, and diagnostic-quality radiographs at four time points—preoperatively, immediately postoperatively (within 7 days), at 1–2 months postoperatively, and at ≥6 months postoperatively. A case was excluded when any of the following applied: radiographic follow-up was incomplete, radiographic positioning was inadequate, intraoperative or perioperative implant failure necessitated revision surgery, or implant failure arose during the follow-up period.

Both stifles were included for five dogs, accounting for 10 of the 30 stifles. As paired stifles come from the same animal, they cannot be treated as statistically independent; they were nonetheless analyzed as separate units, and the resulting potential non-independence is acknowledged as a limitation of this study (see Section 4).

### 2.2. Surgical Procedure

Every TPLO was carried out by the same board-certified veterinary surgeon following a standardized protocol [3,18]. A radial cut was made in the proximal tibia, and the plateau was rotated until a target TPA of roughly 5–6° was obtained. The osteotomy was stabilized with locking TPLO plates (Jeil Medical Corp., Seoul, Republic of Korea), as locking screws have been reported to limit post-TPLO TPA change [19].

### 2.3. Radiographic Evaluation and TPA Measurement

At every time point, standard mediolateral radiographs covering both the stifle and the tarsal joint were acquired, with care taken to position the limb correctly and to superimpose the femoral condyles [8,10].

TPA was determined with digital orthopedic planning software (vPOP-pro version 3.0, VetSOS Education Ltd., Exeter, UK) and defined as the angle formed between the tibial plateau line and a line drawn perpendicular to the mechanical tibial axis, which connects the intercondylar eminence to the center of the talus [3,10]. A single observer measured each radiograph three times, and the average was taken to minimize intraobserver variability. ΔTPA was calculated as ΔTPA = TPA (≥6 months) − TPA (immediate postoperative). For the ≥6 month assessment, the most recent follow-up radiograph available for each stifle was used.

### 2.4. Statistical Analysis

Time-point differences in TPA were tested with paired *t*-tests, while the relationship between body weight and ΔTPA was assessed by Pearson correlation, with significance set at *p* < 0.05. The ≥6 month follow-up threshold was chosen so that assessment fell well past the radiographic bone-healing phase. Parametric tests served as the primary analyses on the basis of the central limit theorem at *n* = 30, the *t*-test being reasonably robust to departures from normality once the sample reaches approximately 30 [20]. All calculations used GraphPad Prism 10 (GraphPad Software, San Diego, CA, USA). Every analysis treated bilateral cases as separate stifle units; to check the resulting non-independence, a sensitivity analysis retained a single stifle per bilaterally operated dog (the first-operated side), leaving 25 independent stifles, and the paired ΔTPA test and body-weight correlation were rerun. The effect of concurrent medial patellar luxation (MPL) was examined by comparing ΔTPA in stifles with and without concurrent MPL repair using an independent-sample (Welch) *t*-test, and the paired ΔTPA analysis was also repeated in the MPL-free subgroup (*n* = 19). As objective functional outcomes (e.g., force-plate gait analysis or validated lameness scoring) were not available, all analyses and outcomes were confined to radiographic measurements.

## 3. Results

### 3.1. Study Population

A total of 30 stifles from 25 client-owned small-breed dogs were enrolled. The cohort comprised 16 spayed females and 9 castrated males (*n* = 25 dogs). Represented breeds were Maltese (*n* = 9), Bichon Frise (*n* = 4), Mixed breed (*n* = 3), Poodle (*n* = 2), Pomeranian (*n* = 2), Cavalier King Charles Spaniel (*n* = 1), Chihuahua (*n* = 1), Corgi (*n* = 1), Shiba Inu (*n* = 1), and Spitz (*n* = 1). Per-stifle body weight spanned 2.56 to 9.46 kg (mean: 6.58 ± 2.24 kg), and five dogs each contributed both stifles (10 stifles, 33.3%).

Concurrent medial patellar luxation (MPL) repair accompanied 11 stifles (36.7%)—bilateral MPL in 7, left MPL in 2, and right MPL in 2. Left-sided procedures accounted for 21 of the 30 stifles (70.0%) and right-sided for 9 (30.0%). The mean follow-up from surgery to the last radiographic evaluation was 12.6 ± 6.8 months (median: 10.3 months; range: 6.0–37.0 months; *n* = 30 stifles). Table 1 provides the individual data for all stifles.

### 3.2. Changes in Tibial Plateau Angle

**Figure 1:**
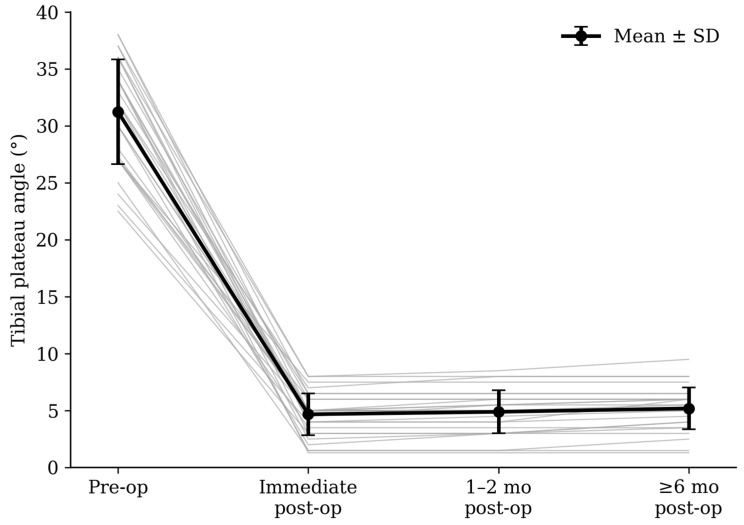
Individual TPA trajectories following TPLO in small-breed dogs (*n* = 30 stifles). Thin gray lines represent individual stifles; the bold line shows the mean ± SD at each time point.

Table 2 summarizes the TPA measurements. The mean preoperative TPA of 31.23 ± 4.60° (range: 22.5–38.0°) fell sharply to 4.68 ± 1.84° right after surgery, a mean reduction of 26.55 ± 4.59° (*t*(29) = 31.714, *p* < 0.001). A postoperative TPA of ≤5° was reached in 22 of 30 stifles (73.3%) and ≤6° in 24 of 30 stifles (80.0%). Mean TPA was 4.89 ± 1.89° at 1–2 months and 5.19 ± 1.83° at ≥6 months. Figure 1 depicts the individual TPA trajectories.

The mean ΔTPA was 0.52 ± 0.64° (95% CI: 0.28–0.75°; *t*(29) = −4.447, *p* < 0.001), and the 1–2 month versus ≥6 month comparison likewise showed a significant increase (*t*(29) = −3.393, *p* = 0.002).

### 3.3. Individual Case Analysis

Twenty-seven stifles (90.0%) exhibited a ΔTPA within 1°, sixteen of them with no change whatsoever. A 1.5° increase was seen in one stifle (3.3%), and two (6.7%) had a ΔTPA of 2.0°.

### 3.4. Correlation Analysis

**Figure 2:**
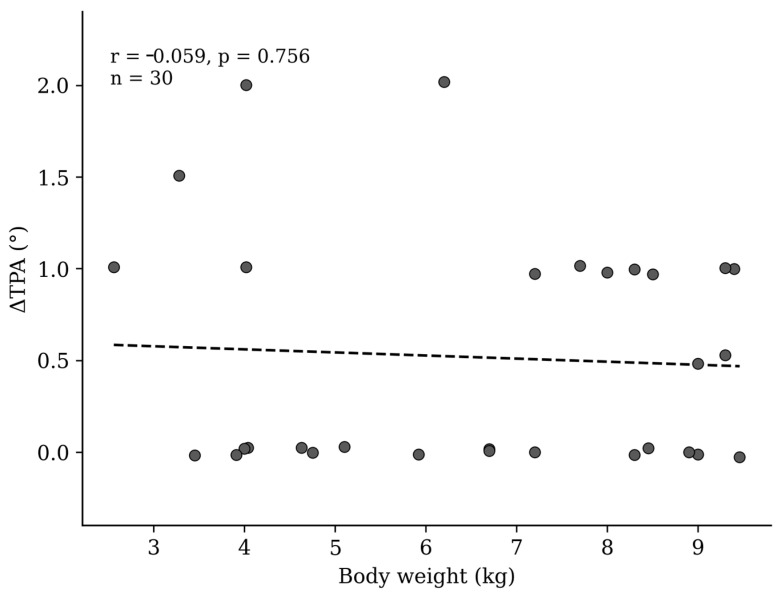
Correlation between body weight and TPA change (r = −0.059, *p* = 0.756; *n* = 30). Each circle represents an individual stifle (*n* = 30). The dashed line represents the linear regression fit.

Pearson correlation returned r = −0.059 (*p* = 0.756), pointing to no meaningful linear association between body weight and ΔTPA in this cohort (Figure 2, Table 3).

### 3.5. Sensitivity and Subgroup Analyses

Limiting the dataset to one stifle per dog (*n* = 25) gave a mean ΔTPA of 0.56 ± 0.67° (95% CI: 0.29–0.84°; *t*(24) = 4.20, *p* < 0.001), with body weight still uncorrelated with ΔTPA (r = −0.034, *p* = 0.870), in line with the full-cohort finding. In the 19 stifles without concurrent MPL repair, the mean ΔTPA was 0.40 ± 0.49° (95% CI: 0.16–0.63°; *t*(18) = 3.53, *p* = 0.002), while the 11 stifles that underwent concurrent MPL repair had a mean ΔTPA of 0.73 ± 0.82° (95% CI: 0.18–1.28°; *t*(10) = 2.95, *p* = 0.015). This difference between the MPL and non-MPL groups did not reach statistical significance (Welch *t*(14.2) = 1.23, *p* = 0.239). Throughout these analyses, the mean ΔTPA stayed below the 1.5° intraobserver measurement error reported by Fettig et al. [8].

## 4. Discussion

In every dog, TPLO achieved a substantial reduction in TPA, and the corrected angle stayed largely unchanged across a mean follow-up of 12.6 ± 6.8 months (median: 10.3 months; range: 6.0–37.0 months), yielding informative long-term radiographic data for small-breed dogs. At 0.52°, the mean ΔTPA was numerically small and is most appropriately judged against established measurement error [8,10]. The mean preoperative TPA in this cohort (31.23°) was somewhat above the range usually reported for the general canine population, in line with earlier observations that small-breed dogs tend to show relatively high TPA values [16].

This result must be interpreted in the context of known measurement error. Because TPA measurements in the present study were performed by a single observer, the most directly comparable benchmark is the intraobserver standard deviation of 1.5° reported by Fettig et al. [8]. The observed mean ΔTPA of 0.52° is substantially smaller than this value, as well as below the typical 1–2° difference introduced by positioning errors [9]. For context, the interobserver standard deviation of 0.8° reported by Fettig et al. [8] is also exceeded, although this value reflects between-observer variation in a multi-observer setting and is not directly applicable to a single-observer design. These comparisons are summarized in Table 4. Together, they suggest that the observed difference is likely attributable to measurement variability rather than true biological change [8,10]. Statistical significance in this analysis is best explained by the narrow within-subject variance in our cohort; with variance this small, trivial differences can still produce significant *p*-values that hold no real biological weight. Notably, all observed ΔTPA values were ≥0, which is not fully consistent with purely random measurement error and may indicate a subtle systematic increase. However, the magnitude remained within reported measurement variability, so it is unlikely to be radiographically meaningful.

A prospective study of 60 dogs by Volz et al. [13] reported a comparable mean TPA shift of 0.22 ± 0.75° at 6 months and detected no relationship between TPA change and functional recovery. The TPA changes in our cohort under 10 kg were similarly minimal in magnitude. However, because the present study did not assess limb function or clinical outcome, our data speak to radiographic stability rather than to clinical relevance, and the parallel with Volz et al. is accordingly limited to the radiographic findings.

Pronounced mean increases in TPA have been reported previously, mainly in medium- and large-breed populations [11,12]. The much smaller changes seen here support the view that most of the variation falls within measurement error rather than representing genuine bony remodeling. Our ≥6 month follow-up window extends well beyond the period of active bone healing. Investigations using shorter intervals—such as the 28–65 day window of Moeller et al. [11]—may instead reflect mechanical settling during early healing rather than true long-term change.

At 4.68°, the mean postoperative TPA in our cohort lies close to the conventional surgical target of approximately 5–6°. Although satisfactory clinical outcomes have been reported even with postoperative TPA values as high as 14° in Labrador Retrievers [21]—suggesting that functional recovery is fairly tolerant of the exact angle obtained—radiographic stability appears more dependent on it. A lower postoperative TPA has been associated with reduced long-term TPA variation [12] and with more symmetrical hindlimb gait [13]. The low postoperative TPA attained in this cohort may thus have contributed to the radiographic stability we observed.

While a lower body mass might be anticipated to lessen the mechanical forces acting across the stifle and so help maintain the corrected TPA, our within-cohort analysis offered no direct evidence for this mechanism, and verifying any load-related effect would require dedicated biomechanical testing. The lack of a meaningful correlation between body weight and ΔTPA (r = −0.059, *p* = 0.756) further suggests that body weight was not a driver of postoperative TPA change in this group. Taken together, the small magnitude of ΔTPA and the absence of a body-weight relationship strengthen the interpretation that the observed differences stem from measurement variability rather than load-related remodeling.

Several limitations apply to this work. Its retrospective nature restricts how well confounders can be controlled, and requiring complete radiographic follow-up, together with the exclusion of cases with implant failure or revision surgery, may have biased inclusion toward cases with smoother postoperative courses. Postoperative management could not be fully standardized. MPL repair was concurrently performed in 36.7% of stifles, potentially altering joint mechanics in those cases. To address this, we compared ΔTPA between stifles with and without concurrent MPL repair (Section 3.5); the difference was not statistically significant (*p* = 0.239), and stability was preserved in the MPL-free subgroup, indicating that concurrent MPL repair did not materially affect the radiographic stability observed, although the modest subgroup sizes limit firm conclusions. TPA measurements were performed by a single observer; although each image was measured three times and mean values were used, formal repeatability statistics such as the intraclass correlation coefficient (ICC) were not calculated for this cohort, representing a limitation when interpreting the observed ΔTPA relative to published measurement error values [8,10]. Five dogs contributed bilateral cases (10 stifles), introducing statistical non-independence; to mitigate this, we performed a sensitivity analysis restricted to one stifle per dog, which yielded essentially the same result (Section 3.5). Larger future studies could further address within-dog clustering using mixed-effects models or generalized estimating equations. In addition, three paired *t*-tests were performed on TPA comparisons without formal correction for multiple testing; however, our overall interpretation does not rely on statistical significance per se, but rather on the magnitude of ΔTPA falling within reported measurement error, so the conclusions remain robust regardless of multiple-testing adjustment. We also did not include objective functional outcomes such as force-plate gait analysis or validated lameness scoring, so the clinical impact of even minor TPA changes cannot be ruled out from our data alone. Future work should ideally use multiple observers, objective gait measures, and longer follow-up.

## 5. Conclusions

TPLO produced a substantial TPA reduction in small-breed dogs, and the correction held up over at least 6 months of follow-up. The 0.52° ΔTPA recorded here (95% CI: 0.28–0.75°) sits below the 1.5° intraobserver measurement variability reported in the literature [8]—the most relevant benchmark given the single-observer design—and aligns with the prospective findings of Volz et al. [13]. Overall, the small TPA changes observed here are more likely a reflection of measurement variability than actual bony remodeling, and this stability was confirmed in a one-stifle-per-dog sensitivity analysis and in the subgroup without concurrent MPL. Because functional outcomes were not evaluated and cases requiring revision were excluded, these conclusions are limited to radiographic angular stability; within that scope, TPLO provided durable TPA correction in small-breed dogs.

## Figures and Tables

**Table 1:** Individual case data for all 30 stifles.

Case	Breed	Sex	BW (kg)	Pre-Op TPA (°)	Post-Op TPA (°)	1–2 Mo TPA (°)	≥6 Mo TPA (°)	ΔTPA (°)	Side	MPL
1	Chihuahua	CM	2.56	24.0	5.0	5.5	6.0	+1.0	Lt	None
2	Maltese	SF	4.04	37.0	8.0	8.0	8.0	0.0	Lt	None
3	Mixed breed	CM	7.70	34.0	5.0	6.0	6.0	+1.0	Lt	None
4	Spitz	CM	9.00	23.0	4.0	4.0	4.5	+0.5	Rt	None
5	Spitz	CM	9.00	25.0	1.5	1.5	1.5	0.0	Lt	None
6	Shiba Inu	SF	8.45	27.3	5.0	5.0	5.0	0.0	Lt	None
7	Maltese	CM	8.50	31.0	5.0	5.5	6.0	+1.0	Lt	None
8	Bichon Frise	SF	4.00	30.0	5.0	5.0	5.0	0.0	Lt	Bilateral MPL
9	Poodle	CM	6.20	30.0	2.0	3.0	4.0	+2.0	Lt	Left MPL
10	Bichon Frise	CM	9.40	36.0	4.0	4.5	5.0	+1.0	Rt	None
11	Maltese	SF	5.92	22.5	3.0	3.0	3.0	0.0	Lt	None
12	Maltese	SF	3.91	34.0	1.3	1.3	1.3	0.0	Lt	None
13	Cavalier KCS	SF	8.30	27.0	7.5	7.5	7.5	0.0	Rt	None
14	Cavalier KCS	SF	8.30	32.0	1.5	1.5	2.5	+1.0	Lt	None
15	Bichon Frise	CM	8.90	27.0	5.0	5.0	5.0	0.0	Lt	None
16	Corgi	SF	9.30	34.0	4.5	5.5	5.5	+1.0	Rt	Right MPL
17	Corgi	SF	9.30	32.0	5.0	5.0	5.5	+0.5	Lt	Left MPL
18	Mixed breed	SF	6.70	38.0	6.5	6.5	6.5	0.0	Lt	None
19	Mixed breed	SF	6.70	38.0	5.0	5.0	5.0	0.0	Rt	None
20	Poodle	SF	5.10	27.0	3.5	3.5	3.5	0.0	Lt	None
21	Pomeranian	SF	3.45	28.0	5.0	5.0	5.0	0.0	Rt	Bilateral MPL
22	Maltese	SF	8.00	34.0	2.5	3.0	3.5	+1.0	Lt	None
23	Maltese	SF	3.28	36.0	8.0	8.5	9.5	+1.5	Lt	Bilateral MPL
24	Mixed breed	CM	9.46	30.0	5.0	5.0	5.0	0.0	Rt	Right MPL
25	Bichon Frise	SF	7.20	32.0	7.0	8.0	8.0	+1.0	Lt	None
26	Bichon Frise	SF	7.20	33.0	6.5	6.5	6.5	0.0	Rt	None
27	Pomeranian	SF	4.75	27.0	6.0	6.0	6.0	0.0	Lt	Bilateral MPL
28	Maltese	CM	4.63	35.0	6.0	6.0	6.0	0.0	Lt	Bilateral MPL
29	Maltese	SF	4.02	36.0	3.0	3.0	4.0	+1.0	Lt	Bilateral MPL
30	Maltese	SF	4.02	37.0	4.0	4.0	6.0	+2.0	Rt	Bilateral MPL

Cavalier KCS = Cavalier King Charles Spaniel; BW = body weight; ΔTPA = TPA change from immediate post-op to ≥6 months; MPL = medial patellar luxation; CM = castrated male; SF = spayed female. Bilateral cases (same individual, both stifles): Cases 4 and 5; 13 and 14; 16 and 17; 18 and 19; 25 and 26. Cases 29 and 30 share breed, sex, and body weight by coincidence but represent different individuals.

**Table 2:** TPA measurements at each time point.

Statistic	Preoperative	Immediate Post-Op	1–2 Months Post-Op	≥6 Months Post-Op
Mean TPA (°)	31.23	4.68	4.89	5.19
SD (°)	4.60	1.84	1.89	1.83
Range (°)	22.5–38.0	1.3–8.0	1.3–8.5	1.3–9.5

*n* = 30 stifles; SD = standard deviation; TPA = tibial plateau angle.

**Table 3:** Summary of statistical analyses.

Analysis	Statistic	*p*-Value
TPA: pre-op vs. immediate post-op	t(29) = 31.714	<0.001
ΔTPA: immediate post-op vs. ≥6 months	t(29) = −4.447	<0.001
TPA: 1–2 months vs. ≥6 months	t(29) = −3.393	0.002
Body weight vs. ΔTPA (Pearson)	r = −0.059	0.756

TPA = tibial plateau angle; ΔTPA = change in TPA; *p*-values calculated using paired *t*-test (TPA comparisons) and Pearson correlation (body weight vs. ΔTPA).

**Table 4:** Comparison of observed ΔTPA with reported measurement errors and prior findings.

Item	Value (°)	Source
Mean ΔTPA (present study)	0.52 ± 0.64	Present study
Intraobserver SD (primary comparator)	1.5	Fettig et al. [8]
Positioning error	1–2	Reif et al. [9]
ΔTPA at 6 months	0.22 ± 0.75	Volz et al. [13]
Interobserver SD (reference only)	0.8	Fettig et al. [8]

SD = standard deviation; ΔTPA = change in tibial plateau angle from immediate post-op to ≥6 months. The mean ΔTPA (0.52°) is smaller than the intraobserver SD (1.5°), the most directly comparable benchmark for the single-observer design used here. The interobserver SD (0.8°) reflects between-observer variation and is presented for reference only.

## Data Availability

The data presented in this study are available on request from the corresponding author.

## Abbreviations

The following abbreviations are used in this manuscript:

TPLO	Tibial plateau leveling osteotomy
TPA	Tibial plateau angle
ΔTPA	Change in tibial plateau angle
CCLD	Cranial cruciate ligament disease
MPL	Medial patellar luxation
CI	Confidence interval
SD	Standard deviation
CM	Castrated male
SF	Spayed female
